# Association of Fcγ Receptor IIB Polymorphism with Cryptococcal Meningitis in HIV-Uninfected Chinese Patients

**DOI:** 10.1371/journal.pone.0042439

**Published:** 2012-08-03

**Authors:** Xiu-Ping Hu, Ji-Qin Wu, Li-Ping Zhu, Xuan Wang, Bin Xu, Rui-Ying Wang, Xue-Ting Ou, Xin-Hua Weng

**Affiliations:** Department of Infectious Diseases, Huashan Hospital, Fudan University, Shanghai, China; Sudbury Regional Hospital, Canada

## Abstract

**Background:**

As important regulators of the immune system, the human Fcγ receptors (FcγRs) have been demonstrated to play important roles in the pathogenesis of various infectious diseases. The aim of the present study was to identify the association between *FCGR* polymorphisms and cryptococcal meningitis.

**Methodology/Principal Findings:**

In this case control genetic association study, we genotyped four functional polymorphisms in low-affinity FcγRs, including *FCGR2A* 131H/R, *FCGR3A* 158F/V, *FCGR3B* NA1/NA2, and *FCGR2B* 232I/T, in 117 patients with cryptococcal meningitis and 190 healthy controls by multiplex SNaPshot technology. Among the 117 patients with cryptococcal meningitis, 59 had predisposing factors. In patients with cryptococcal meningitis, the *FCGR2B* 232I/I genotype was over-presented (OR = 1.652, 95% CI [1.02–2.67]; P = 0.039) and the *FCGR2B* 232I/T genotype was under-presented (OR = 0.542, 95% CI [0.33–0.90]; P = 0.016) in comparison with control group. In cryptococcal meningitis patients without predisposing factors, *FCGR2B* 232I/I genotype was also more frequently detected (OR = 1.958, 95% CI [1.05–3.66]; P = 0.033), and the *FCGR2B* 232I/T genotype was also less frequently detected (OR = 0.467, 95% CI [0.24–0.91]; P = 0.023) than in controls. No significant difference was found among *FCGR2A* 131H/R, *FCGR3A* 158F/V, and *FCGR3B* NA1/NA2 genotype frequencies between patients and controls.

**Conclusion/Significance:**

We found for the first time associations between cryptococcal meningitis and *FCGR2B* 232I/T genotypes, which suggested that FcγRIIB might play an important role in the central nervous system infection by *Cryptococcus* in HIV-uninfected individuals.

## Introduction

Cryptococcal meningitis is the most common opportunistic fungal infection of the central nervous system in AIDS patients. Among HIV-uninfected patients, several predisposing factors for cryptococcal meningitis such as corticosteroid medication, solid organ transplantation and malignancy, etc, have been indentified. Yet cryptococcal infections in apparently healthy individuals are also increasingly being reported, especially from Asian data [1]–[3]. Our previous study has demonstrated an association between mannose-binding lectin (MBL) genetic deficiency and cryptococcal meningitis in HIV-uninfected patients [4]. However, MBL deficiency was present in only 21% of the cases, and for the remaining 79% of patients the underlying mechanism for susceptibility remained unclear.

Fc gamma receptors (FcγRs) mediate a variety of immune responses after binding to IgG-opsonized pathogens or immune complexes, and therefore act as immune regulators in both autoimmune and infectious diseases [5]–[9]. According to their affinity to IgG, FcγRs are categorized into high-affinity and low-affinity receptors. FcγRI is the only known high-affinity receptor. Low-affinity FcγRs which include FcγRIIA, FcγRIIB, FcγRIIC, FcγRIIIA, and FcγRIIIB, are encoded by *FCGR2A*, *FCGR2B*, *FCGR2C*, *FCGR3A*, and *FCGR3B* genes, respectively.

*FCGR* polymorphisms had been associated with the susceptibility and severity of various infections. *FCGR2A* 131R/R had been reported to attribute to the susceptibility of meningococcal infection, community-acquired pneumonia (CAP) caused by *Haemophilus influenza,* and the development of severe malaria [10]–[12]. *FCGR2A* 131H/H was reported to contribute to higher risk of bacteremia in pneumococcal CAP patients [13]. Another study showed that HIV-infected patients with *FCGR2A* 131R/R genotype progressed to a low CD4^+^ cell count at a faster rate, but conversely in individuals carried *FCGR2A* 131H/H there was an increased risk of *Pneumocystis jiroveci* pneumonia [14]. *FCGR3A* 158F/V gene polymorphism was not associated with progression of HIV infection, but predicted the risk of Kaposi’s sarcoma [14]. A study on infections during induction chemotherapy found that *FCGR2A* 131H/H was associated with a decreased risk of pneumonia, *FCGR3B* NA1/NA1 associated with infections, and *FCGR3A* polymorphisms not associated with infections [15]. Sadki et al. investigated the influence of *FCGR3A* 158V/F and *FCGR2A* 131H/R polymorphisms on susceptibility to pulmonary tuberculosis in the Moroccan population but no association was found [16]. A study in East Africa found that the *FCGR2B* 232T/T genotype provided protectiveness for children against severe malaria [17].

A previous study by Meletiadis et al. investigated *FCGR* polymorphisms in patients with cryptococcosis, and found that *FCGR2A* 131R/R and *FCGR3A* 158V/V were over-presented, and *FCGR3B* NA2/NA2 was under-presented in patients with cryptococcosis [18]. The purpose of this study was to investigate *FCGR* polymorphisms in our series of patients to further verify the association between *FCGR* and cryptococcal meningitis.

## Results

### Demographic Characteristics

A total of 117 HIV-uninfected patients with cryptococcal meningitis were included. Subjects from both the patient and control groups were of Chinese Han ethnicity. Clinical information and predisposing factors of the patients are summarized in Table 1. Of the 190 healthy control subjects, 111 were male (58.4%). The median age of the control subjects was 44 years (range, 12–79 years).

**Table 1:** Clinical information and predisposing factors in 117 patients with cryptococcal meningitis.

Characteristics	N (%)/median (range)
Male	74 (63.2)
Age (years)	45 (14–78)
Confirmed cases	101 (86.3)
Probable cases	16 (13.7)
Predisposing factors^a^	59 (50.4)
Autoimmune diseases	18 (15.4)
Diabetes mellitus	13 (11.1)
Liver cirrhosis	6 (5.1)
Chronic kidney diseases	5 (4.3)
Solid malignancies	2 (1.7)
Kidney transplantation	2 (1.7)
Corticosteroids^b^	21 (17.9)
Immunosuppression^c^	12 (10.3)
Idiopathic CD4^+^ T lymphocytopenia	13 (11.1)
Severe cryptococcal meningitis^d^	35 (29.9)
Disturbance of consciousness	31 (26.5)
Cerebral herniation	9 (7.7)
Death	5 (4.3)

NOTE: ^a^Predisposing factors including immunocompromising diseases (liver cirrhosis, chronic kidney diseases, autoimmune diseases, malignancies, solid organ transplantation, diabetes mellitus), and corticosteroid or immunosuppressive medications, and idiopathic CD4^+^ T lymphocytopenia. Some patients had more than one predisposing factors.

b Defined as receiving prednisone of a mean minimum dose of 0.3 mg/kg/day, or equivalent doses of other corticosteroids for >3 weeks.

c Immunosuppressive agents including cyclosporine, tripterygium glycosides, vincristine, etc.

d Patients with one or more conditions including disturbance of consciousness, cerebral herniation, and death were classified as severe cases.

### Genotype Distribution

Two samples failed genotyping of *FCGR3A* and 2 samples failed in genotyping of *FCGR2B*. Allele distributions of the tested *FCGR* genes in the control group were in Hardy-Weinberg equilibrium. The frequencies of *FCGR2A*, *FCGR3A*, *FCGR3B* and *FCGR2B* genotypes were shown in Table 2. An association was found between *FCGR2B* 232I/T genotypes and cryptococcal meningitis based on dominant and over-dominant model. The *FCGR2B* 232I/I genotype was over-presented (OR = 1.652, 95% CI [1.02–2.67]; P = 0.039) and the *FCGR2B* 232I/T genotype was under-presented (OR = 0.542, 95% CI [0.33–0.90]; P = 0.016) in patients with cryptococcal meningitis in comparison with controls. No significant difference was found in the distribution of *FCGR2A* 131H/R, *FCGR3A* 158 F/V and *FCGR3B* NA1/NA2 genotypes.

**Table 2:** Distribution of *FCGR2A*, *FCGR3A*, *FCGR3B, FCGR2B* genotypes in patients with cryptococcal meningitis and controls^a^.

Genotypes	All patients (117)				Patients without predisposing factors (58)				Controls (190)	
	N	%	P	OR (95% CI)	N	%	P	OR (95% CI)	N	%
*FCGR2A*										
131H/H (Dominant b)	47	40	0.335	0.795 (0.50–1.27)	28	48	0.740	1.105 (0.61–1.99)	87	46
131H/R (Over-dominant^b^)	48	41	0.859	1.043 (0.65–1.67)	23	40	0.963	0.986 (0.54–1.80)	76	40
131R/R (Recessive b)	22	19	0.286	1.398 (0.75–2.59)	7	12	0.678	0.829 (0.34–2.02)	27	14
131H (Allelic^b^)	142	61	0.201	0.803 (0.57–1.13)	79	75	0.644	1.110 (0.71–1.73)	250	66
*FCGR3A*										
158F/F (Dominant)	52	45	0.889	0.967 (0.61–1.54)	28	48	0.687	1.129 (0.63–2.03)	86	45
158F/V (Over-dominant)	54	46	0.740	1.082 (0.68–1.72)	22	38	0.397	0.771 (0.42–1.41)	84	44
158V/V (Recessive)	11	9	0.751	0.882 (0.41–1.91)	8	14	0.491	1.360 (0.57–3.27)	20	11
158F (Allelic)	158	68	0.969	1.007 (0.71–1.43)	76	74	0.980	0.994 (0.64–1.55)	256	67
*FCGR3B*										
NA1/NA1 (Dominant)	34	29	0.852	0.953 (0.57–1.58)	15	26	0.576	0.827 (0.43–1.61)	57	30
NA1/NA2 (Over-dominant)	73	63	0.276	1.301 (0.81–2.09)	38	67	0.176	1.533 (0.82–2.85)	107	57
NA2/NA2 (Recessive)	9	8	0.178	0.578 (0.26–1.29)	4	7	0.341^c^	0.519 (0.17–1.56)	24	13
NA1/NA3^d^	0	0	–	–	0	0	–	–	1	–
NA1 (Allelic)	141	61	0.626	1.087 (0.78–1.52)	68	65	0.868	1.037 (0.68–1.60)	221	58
*FCGR2B*										
232I/I (Dominant)	75	65	0.039*	1.652 (1.02–2.67)	40	69	0.033*	1.958 (1.05–3.66)	101	53
232I/T (Over-dominant)	31	27	0.016*	0.542 (0.33–0.90)	14	24	0.023*	0.467 (0.24–0.91)	77	40
232T/T (Recessive)	9	8	0.614	1.259 (0.51–3.09)	4	7	1.000^c^	1.099 (0.34–3.55)	12	7
232I (Allelic)	131	79	0.128	1.352 (0.92–1.99)	94	89	0.097	1.547 (0.92–2.59)	279	73

NOTE: ^a^The patient group included 101 confirmed cases and 16 probable cases of cryptococcal meningitis, among which 59 were with predisposing factors and 58 were apparently healthy.

b Dominant: heterozygotes and homozygotes for mutant allele vs. homozygotes for wildtype allele. Over-dominant: homozygotes for mutant and wildtype allele vs. heterozygotes. Recessive: homozygotes for mutant allele vs. heterozygotes and homozygotes for wildtype allele. Allelic: mutant alleles vs. wildtype alleles.

c Analyzed by Fisher’s exact test. The other data were all analyzed with 2×2 χ^2^ test.

d There was a haplotype of *FCGR3B* NA (141G-147C-227G-277A-349A) in controls which has not been reported in previous studies. We defined it as NA3.

* P<0.05.

We further compared the genotype distribution of *FCGR2A*, *FCGR3A*, *FCGR3B* and *FCGR2B* between the 58 patients without predisposing condition and controls. Similar to results from the overall patient group, associations were also found between *FCGR2B* 232I/T genotypes and cryptococcal meningitis based on dominant and over-dominant model. Specifically, *FCGR2B* 232I/I genotype was also more frequently detected (OR = 1.958, 95% CI [1.05–3.66]; P = 0.033), and *FCGR2B* 232I/T genotype was also less frequently detected (OR = 0.467, 95% CI [0.24–0.91]; P = 0.023) in patients without predisposing factor than in controls. For the genotype distribution of other polymorphisms (*FCGR2A* 131H/R, *FCGR3A* 158 F/V and *FCGR3B* NA1/NA2), there was also no significant difference between patients and controls.

## Discussion

The distribution of *FCGR* polymorphisms has been reported to exhibit substantial inter-ethnic variation. According to our population, frequencies of *FCGR2A* 131R/R, *FCGR3B* NA2/NA2, and *FCGR2B* 232T/T in all subjects were 16%, 11%, and 7% respectively, similar to those reported among other Asian populations (which ranged 9–14%, 11–21%, and 5–11%) [19]–[26]. Frequencies of these genotypes in Caucasian population were reported to be 19–34%, 38–43%, and 1–3% [18], [23], [27]–[30], which were different from our data. There seems no marked difference in the distribution of *FCGR3A* 158F/V genotypes between the Asian and Caucasian populations [18], [21], [23], [31], [32].

The four polymorphisms of low-affinity receptors genotyped in our study have each been demonstrated to affect functions of their encoded receptors. In *FCGR2A,* the G>A SNP at amino acid position 131 results in a histidine (H) to arginine (R) change (*FCGR2A* 131H/R), resulting in reduced affinity of the correspondent receptor to IgG2 [33], [34]. The T>G SNP at position 158 of *FCGR3A* causes a phenylalanine (F) to valine (V) substitution (*FCGR23A* 131F/V) and *FCGR3A* 158V/V encoded receptors show higher affinity to IgG1 and IgG3 [35], [36]. In the *FCGR3B* gene, five nucleotides (141,147,227,277 and 349) are combined to form two main haplotypes termed *FCGR3B* NA1 and *FCGR3B* NA2, and receptor encoded by *FCG3B* NA1 haplotype binds to IgG1 and IgG3 more easily [37]. Finally, *FCGR2B* 232I/T causes an isoleucine (I) to threonine (T) substitution in the transmembrane domain [22], [38] and receptors encoded by *FCGR2B* 232T are unable to interact with activating receptors [39].

Although *FCGR* polymorphisms have been demonstrated to be associated with susceptibility and severity of numerous infections, there has only been one previous genetic association study on the relationship of *FCGR* genotypes and cryptococcosis [18]. Meletiadis and colleagues genotyped *FCGR2A* 131H/R, *FCGR3A* 158F/V and *FCGR3B* NA1/NA2 in 103 cryptococcosis patients and 395 healthy controls. They found that in patients with cryptococcosis *FCGR2A* 131R/R and *FCGR3A* 158V/V were over-presented (P-values were 0.04 and 0.04), while *FCGR3B* NA2/NA2 was under-presented (P-value was 0.04).

In our study, we found for the first time that cryptococcal meningitis was associated with the *FCGR2B* 232I/T genotypes, which was not reported in Metediatis’ study. As the only known inhibitory FcγR, FcγRIIB has an immunoreceptor tyrosine-based inhibitory motif (ITIM) in its cytoplasmic domain, and thus it plays an important role in regulating the immune system [40]. *FCGR2B* 232I/T is located in the transmembrane domain, and the FcγRIIB receptors encoded by *FCGR2B* 232T are unable to interact with activating receptors and exert inhibitory activity [38]. Published data have suggested the mutation genotype *FCGR2B* 232T/T to be a susceptible genotype for systemic lupus erythematosus [17], [22], [32], and this genotype also provided protective effect for severe malaria in East African children [17]. The role of FcγRIIB in cryptococcal infection is still not very clear. Like the activatory FcγRs, FcγRIIB can also recognize the major component of the capsule of *C. neoformans*, glucuronoxylomannan (GXM). In a previous study by Monari et al., the immunosuppressive effect of GXM was demonstrated to be dependent on FcRγIIB, based on the evidences that FcγRIIB blockade inhibits GXM-induced IL-10 production and induces TNF-α secretion, and that the addition of monoclonal antibody to GXM reverses GXM-induced immunosuppression by shifting recognition from FcγRIIB to FcγRIIA [41]. Another study subsequently demonstrated that GXM triggered NO-induced macrophage apoptosis, which was dependent on FcγRII [42]. These data support that FcγRIIB plays a critical role in the pathogenesis of cryptococcal infection. In our study, it is the *FCGR2B* 232I/T heterozygote instead of the minor homozygote 232T/T that is under-presented in patient group. One study on children with idiopathic thrombocytopenia (ITP) also showed a similar pattern, that the *FCGR2B* 232I/T was less frequently detected in acute ITP in comparison with chronic ITP [27]. The reason for the heterozygotes 232I/T rather than 232T/T under-presenting in our patients and those acute ITP children has not been clarified.

Unlike results from Meletiadis’ study, no association among *FCGR2A* 131H/R, *FCGR3A* 158F/V, *FCGR3B* NA2/NA2 and cryptococcal meningitis was found in our study. The cause for discrepant results may be multifactorial. One was the ethnic differences between the two studies. Subjects in our study were of Chinese Han ethnicity, while the majority of subjects in Meletiadis’ study were Caucasians (60%). As a matter of fact, the *FCGR3A* 158V allele was significantly increased only in patients who were Caucasian in Meletiadis’ study. Secondly, all the cases in our study were diagnosed with cryptococcal meningitis, while some patients from Meletiadis’ study were cryptococcosis without central nervous system involvement. Furthermore, both studies had relatively small sample sizes, which could be underpowered to generate positive results.

In conclusion, our study suggested that FcγRIIB genetic polymorphism may contribute to the susceptibility of cryptococcal meningitis. The overall association is relatively weak, which warrants validation in larger population.

### Ethics Statement

This study was reviewed and approved by the Ethic Committee/Institutional Review Board (HIRB) of Huashan Hospital, Fudan University, and informed written consent was obtained from each participant.

**Table 3:** Product size and primers of eight SNPs in *FCGR*s.

SNP ID	Product size (bp)	PCR primer sequence	Extension primer sequence
*FCGR2A* 131H/R (rs1801274)	587	F:TTGCCTATAAGAGAATGCTCACATCTR:AAGCTCTGGCCCCTACTTGTT	SR: TTTTTTTTTTTTGGAGAAGGTGGGATCCAAA
*FCGR3A* 158F/V (rs396991)	1537	F:GAATTGCCAGGCTGAGCAAR:CAGGCTTTGAAGTCTTTGATGTG	SR: TTTTTTTTTTTTTTTTTTCTGAAGACACATTT TTACTCCCAA
*FCGR2B* 232I/T (rs1050501)	2394	F:CCTCAGCACATATCAGTGGTGGTR:AGCCCAAAGAGAGGGATTCTG	SR: AACAATGGCCGCTACAGCA
*FCGR3B* NA1/NA2 (rs403016,rs447536, rs448740, rs428888, rs2290834)	1413	F:CACATCTATAGCTGTGGATTGAGGTAR:TCCATATGGGGATTCTTGGAA	rs403016SF: TTTTTTTTTTTTTTTTTTTTTTTTCCTGGAGC CTCAATGGTACAGrs447536SR: TTTTTTTTTACTTCAGAGTCACACTGTCCTTCTC rs448740SR: TTTTGGCCTGGCTTGAGATGAGGrs428888SR: TTTTTTTTTTTTTTGCACCTGTACTCTCCACTGTCGTrs2290834SF: TTTTTTTCGGTGCAGCTAGAAGTCCAT

Note: F indicates forward primer, R indicates reverse primer.

## Materials and Methods

### Subjects

A total of 200 volunteers and 117 unrelated patients with proven or probably diagnosed cryptococcal meningitis who were referred to Huashan Hospital, Fudan University, China, from 2001 through 2011 were recruited for the present study. Patients who met at least one of the following criteria were considered as proven cryptococcal meningitis: (1) Isolation of *C. neoformans* from cerebrospinal fluid (CSF) by culture or positive India ink smear, and (2) compatible histopathological findings, which are 5–10 µm encapsulated yeasts observed in brain tissue. Patients who had no microbiological or pathological documentation but present with positive cryptococcal antigen titer (≥1∶10) in CSF and met at least one of the following criteria were regarded as probable cryptococcal meningitis: (1) abnormal laboratory tests or an increased open pressure (≥200 mmH_2_O) of CSF, (2) abnormalities of cranial imaging (Computerized Tomography or Magnetic Resonance Imaging) which could not be explained by other factors, and (3) comorbidities that compromise the host immune system. Cryptococcal antigen was determined using diluted CSF with the Latex-*Cryptococcus* antigen detection system (Immuno-Mycologics). Patients and volunteers were assessed for predisposing factors as follow, immunocompromising diseases (liver cirrhosis, chronic kidney diseases, autoimmune diseases, malignancies, solid organ transplantation) [2], [3], [43], and corticosteroid (at prednisone equivalent dose of >0.3 mg/kg/day of for >3 weeks) or immunosuppressive medications (within 90 days before onset of cryptococcal meningitis) [44], and idiopathic CD4^+^ T lymphocytopenia (unexplained CD4^+^ T lymphocytopenia with CD4^+^ T lymphocyte count <300 cells/mm^3^) [45]. Diabetes mellitus was also included, although this common condition is a controversial predisposing factor [3], [46]. Patients without any of the above mentioned predisposing factors were considered as apparently healthy hosts. Ten volunteers were excluded because of disclosed predisposing conditions, and the remaining 190 healthy volunteers were included in the control group.

### Polymorphisms Selection and Genotyping

Four functional *FCGR* polymorphisms including *FCGR2A* 131H/R, *FCGR3A* 158F/V, *FCGR3B* NA1/NA2, and *FCGR2B* 232I/T were selected for genotyping after literature review of previous studies on association between *FCGR* polymorphisms and infectious diseases [11]–[17].

Venous blood was obtained by venepuncture from each subject. Genomic DNA was extracted using the QIAamp DNA kit (Qiagen, Hilden, Germany) according to manufacturer’s instructions. Genotyping of 8 SNPs in *FCGR*s (Table 3) was performed by multiplex SNaPshot technology using an ABI fluorescence-based assay discrimination method (Applied Biosystems, Foster city, CA, USA), which has been described in detail in previous studies [47], [48]. The multiplex SNaPshot detection of single-base extended probe primers was based on fluorescence and extended length detected by capillary electrophoresis on ABI3130XL Sequencer (Applied Biosystems, Foster City, CA, USA).

Four pairs of primers for PCR amplification including 5 fragments of 587–2394 bp and 8 primers for SNaPshot extension reactions were designed by Primer3 online software (v.0.4.0) (http://frodo.wi.mit.edu/primer3/) according to the reference sequences from dbSNP (http://www.ncbi.nlm.nih.gov/SNP). There were homologous sequences between *FCGR*s, the specificity sequences were checked with the sequence databases using BLAST (http://www.ncbi.nlm.nih.gov/blast/blast.cgi). These sequences were also verified by SNPmasker1.1 (http://bioinfo.ebc.ee/snpmasker) to make sure that the different bases were caused by SNP [49]. And each primer pair was tested for potential primer-dimer and hairpin structures using the AutoDimer software (http://www.cstl.nist.gov/biotech/strbase/AutoDimerHomepage/AutoDimerProgramHomepage.htm). The primers used in this study were listed in Tables 3.

The PCR reactions were performed with 1 µL of DNA and 1 µL multiple PCR primers (the concentration was 1 µM) in a total volume of 20 µL containing 1× HotStarTaq buffer, 2.0 mM Mg^2+^, 0.3 mM dNTP, and 1 U HotStarTaq polymerase (Qiagen, Hilden, Germany). The cycling conditions for *FCGR2A* and *FCGR3A* were 95°C for 2 min, 35 cycles using 96°C for 20 s, 62°C for 2 min, and 72°C for 3 min, then 72°C for 10 min, and finally kept at 4°C. The cycling conditions for *FCGR2B* and *FCGR3B* were 95°C for 2 min, 7 cycles using 96°C for 20 s, 55°C for 2 min, and 72°C for 3 min, then 72°C for 10 min, and finally kept at 4°C. PCR products were then purified (add 1U SAP enzyme to 10 µL PCR products, incubate at 37°C for 1 hour, then, inactivate at 75°C for 15 min).

The extension reaction to identify single nucleotide polymorphisms in the PCR products was performed in a total volume of 10 µL containing 2 µL purified PCR product, 1 µL primer (the concentration was 0.8 µM), 5 µL SNaPshot Multiplex Kit (Applied Biosystems, Foster City, CA, USA), and 2 µL ultrapure water. The cycling conditions for extension were 96°C for 1 min, 28 cycles of 96°C for 10 s, 52°C for 5 s, and 60°C for 30 s, and kept at 4°C. Then each extended product was added to 1 U shrimp alkaline phosphatase, incubated at 37°C for 1 hour, and the enzyme inactivated at 75°C for 15 min. Then, 0.5 µL was added to 0.5 µL Liz120 SIZE STANDARD (Applied Biosystems, Foster City, CA, USA), 9 µL Hi-Di (Applied Biosystems, Foster City, CA, USA), and sequenced by ABI3130XL Sequencer (Applied Biosystems, Foster City, CA, USA). Finally, the primary data was analyzed by GeneMapper 4.0 (Applied Biosystems, Foster City, CA, USA). Genotypes were determined by the type of nucleotide presented at SNP site, which was visualized by one or two different color peaks on the figures.

For quality control, a random sample of 5% of the cases and controls was genotyped twice by different researchers, with a reproducibility of 100%. The minor allele counts were compared with database (http://www.ncbi.nlm.nih.gov/projects/SNP), and the data were matched well. Genotyping was performed blind to group status.

### Statistical Analysis

Dominant, over-dominant, recessive and allelic models were applied for the analysis of genotype distribution. Hardy-Weinberg equilibrium, differences in gene polymorphism distributions between patients and controls were analyzed with 2×2 χ^2^ tests or Fisher’s exact test where appropriate. P-values, odds ratios (ORs) and 95% confidence intervals (CIs) were obtained by SPSS 16.0 for Windows (SPSS, Inc, Chicago, IL). P-values <0.05 were considered statistically significant.
